# A Biomedical Investigation of the Hepatoprotective Effect of *Radix salviae miltiorrhizae* and Network Pharmacology-Based Prediction of the Active Compounds and Molecular Targets

**DOI:** 10.3390/ijms18030620

**Published:** 2017-03-13

**Authors:** Ming Hong, Sha Li, Ning Wang, Hor-Yue Tan, Fan Cheung, Yibin Feng

**Affiliations:** School of Chinese Medicine, The University of Hong Kong, Hong Kong, China; hong1986@connect.hku.hk (M.H.); lishasl0308@163.com (S.L.); ckwang@hku.hk (N.W.); hoeytan@connect.hku.hk (H.-Y.T.); ofcheung@hku.hk (F.C.)

**Keywords:** *Radix salviae miltiorrhizae*, hepatoprotective, antioxidant, network pharmacology

## Abstract

*Radix salviae miltiorrhizae* (Danshen in Chinese), a classic traditional Chinese medicine (TCM) herb, has been used for centuries to treat liver diseases. In this study, the preventive and curative potential of Danshen aqueous extract on acute/chronic alcoholic liver disease (ALD) and non-alcoholic fatty liver disease (NAFLD) was studied. The in vivo results indicated that Danshen could alleviate hepatic inflammation, fatty degeneration, and haptic fibrogenesis in ALD and NAFLD models. In the aspect of mechanism of action, the significant reduction in MDA levels in both ALD and NAFLD models implies the decreased levels of oxidative stress by Danshen. However, Danshen treatment could not activate the internal enzymatic antioxidant system in ALD and NAFLD models. To further explore the hepatoprotective mechanism of Danshen, an in silico-based network pharmacology approach was employed in the present study. The pharmacological network analysis result revealed that six potential active ingredients such as tanshinone iia, salvianolic acid b, and Danshensu may contribute to the hepatoprotective effects of Danshen on ALD and NAFLD. The action mechanism may relate with regulating the intracellular molecular targets such as PPARα, CYP1A2, and MMP2 for regulation of lipid metabolism, antioxidant and anti-fibrogenesis by these potential active ingredients. Our studies suggest that the combination of network pharmacology strategy with in vivo experimental study may provide a forceful tool for exploring the mechanism of action of traditional Chinese medicine (TCM) herb and developing novel bioactive ingredients.

## 1. Introduction

Liver disease is one of the most serious health problems worldwide, affecting more than 10% of the world population [1,2]. Alcoholic liver disease (ALD) and nonalcoholic fatty liver disease (NAFLD) are two common types of liver diseases that represent a major health burden in industrialized countries. ALD is caused by excessive alcohol consumption whereas NAFLD is related with obesity and metabolic disorders, although nonalcoholic steatohepatitis (NASH) has been reported in lean individuals [3]. Both ALD and NAFLD may progress into fibrosis, cirrhosis, and eventually hepatocellular carcinoma as a result of severe and prolonged liver damage [4]. The effect of current synthetic agents in treating ALD and NAFLD is not satisfactory and most of them have undesirable side effects [5]. Thereby, the development of novel agents that can improve the efficacy of ALD and NAFLD prevention and treatment is urgently needed. In recent years, numerous medicinal herbs and phytochemicals have been investigated as complementary and alternative treatments for liver diseases including ALD and NAFLD [2]. However, scientific validation of these herbal medicines’ efficacy is needed.

Danshen is the root part of *Salvia miltiorrhiza* Bunge. According to the TCM theory, Danshen can unblock meridians, dispel stasis and promote regeneration without damaging healthy “Qi”. It is widely indicated for liver disorders caused by blood stasis accumulation and obstruction [6]. The hepatoprotective effects of Danshen have been proved by several clinical trials in NAFLD and hepatocarcinoma patients in recent years [7,8]. Furthermore, modern pharmacological studies also revealed the hepatoprotective effect of Danshen and its active ingredients for liver diseases in vitro and in vivo. For example, the total extracts of Danshen and its components such as tanshinol A, tanshinone I, tanshinone II A, dihydrotanshinono I, neotanshinone A, cryptanshinono, salvianolic acid A and salvianolic acid B were found to exhibit hepatoprotective effects in various hepatic cell lines and animal models against viral hepatitis, paracetamol-induced hepatitis, hepatic fibrosis and hepatocarcinoma [9,10,11,12,13]. Although Danshen has shown potential hepatoprotective effects in several hepatic dysfunctions models, systematical research on its therapeutic effect and the underlying mechanisms on ALD and NAFLD are still limited. Plenty of studies have shown that oxidative stress plays an important role in mediating the pathogenesis of various liver diseases including ALD and NAFLD. Oxidative stress in the liver could induce lipid peroxidation and subsequently results in death of hepatocyte [14,15]. Nature has evolved multiple layers of antioxidant defense in the liver. Small molecular antioxidants such as vitamins E and C offer the first line of defense to scavenge reactive oxygen/nitrogen species (ROS/RNS) directly and thus prevent or delay the initiation of various oxidative stresses. Besides this defense layer, antioxidant enzymes also serve as a major defense to detoxify ROS/RNS into less reactive species. Substantial evidence have shown that Danshen extracts could suppress oxidative stress by increasing the activity of superoxide dismutase (SOD), glutathione peroxidase (GSH-Px) and catalase (CAT) as well as reducing reactive oxygen species (ROS) and malondialdehyde (MDA) levels in mice diabetic renal tissue and rat paracetamol-induced liver injury [16,17,18]. Thus, the aim of the present study was to investigate the potential antioxidant effects of Danshen extract on ALD and NAFLD and explore the underlying mechanisms.

Over the past few decades, there has been a significant decline in the rate of novel phytochemicals translated into effective drugs. Currently, the most important problem for novel drugs development is a lack of therapeutic efficacy in clinical trials, which account for 33% of failures [19]. Thus, to maximize drug efficacy in pharmaceutical development, network pharmacology has been recently introduced to analyze the biological network of drug candidates in order to design a poly-target drug molecule. Network pharmacology is a developing field based in systems pharmacology that looks at the effect of drugs on both interactome and diseasome [20]. The drug-target network plays an important role in understanding the mechanisms of action of approved and experimental drugs. In recent years, network pharmacology has attracted much attention in the field of revealing the molecular mechanisms of TCM herbs for complicated diseases. Zhang et al. explored an integrative platform of TCM network pharmacology and its application on a herbal formula [21]. Li et al. also determined active compounds and action mechanisms of Ge-Gen-Qin-Lian decoction for treatment of type 2 diabetes by using network pharmacology method [22]. Many active chemical compositions of TCM target multiple proteins in the biological network of human diseases. Molecular docking and text mining assays are available for modeling interactions between small molecules and proteins [23,24]. Thus, research into TCM based on network pharmacology, which is a holistic understanding of the molecular mechanisms responsible for the pharmacological effects of herbal medicines, is well worth undertaking.

In the present research, we hypothesized that Danshen may exhibit both preventive and curative effects against alcoholic and non-alcoholic hepatic injuries via relieving oxidative stress. We investigated the biological effects of Danshen on acute/chronic ALD and NAFLD mice models. Then, we utilized computational tools and resources to develop the pharmacological network of Danshen for predicting the potential active ingredients and intracellular molecular targets. The in silico method combined with in vivo studies in this research may decipher the mystery of Danshen and promote TCM-based drug discovery.

## 2. Results

### 2.1. The Hepatoprotective Effect of Danshen on Acute ALD

**Figure 1:**
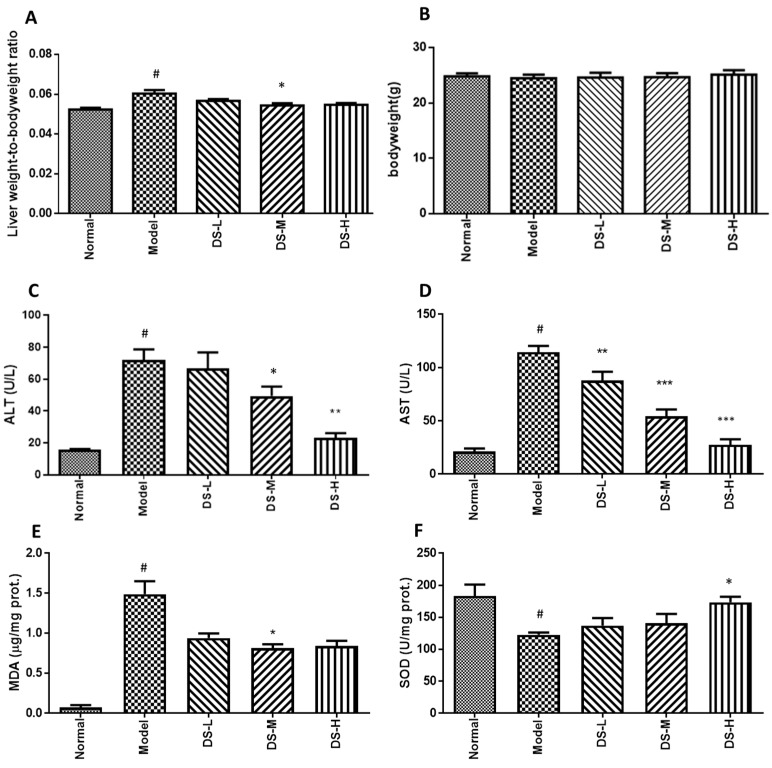

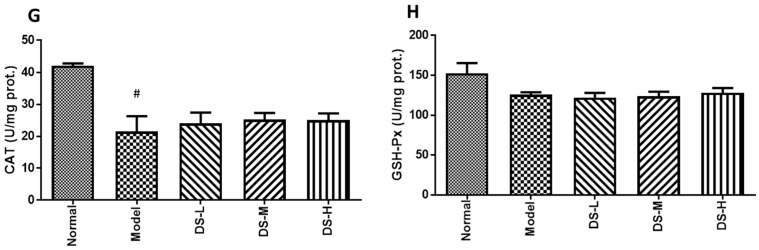
Liver weight to body weight ratio, body weight measurement, and biochemical assays results after Danshen treatment in acute ALD. (**A**,**B**) The liver weight-to-body weight radio and body weight changes; (**C**) serum ALT level; (**D**) serum AST level; (**E**–**H**) the production of MDA and the activity of SOD, CAT, and GSH-Px in liver tissues. DS-L for low dose of Danshen treatment group (0.093 g/kg); DS-M for middle dose of Danshen treatment group (0.28 g/kg); DS-H for high dose of Danshen treatment group (0.84 g/kg). ^#^ *p* < 0.05, when compared with normal group; * *p* < 0.05, ** *p* < 0.01, *** *p* < 0.001 when compared with model group).

**Figure 2:**
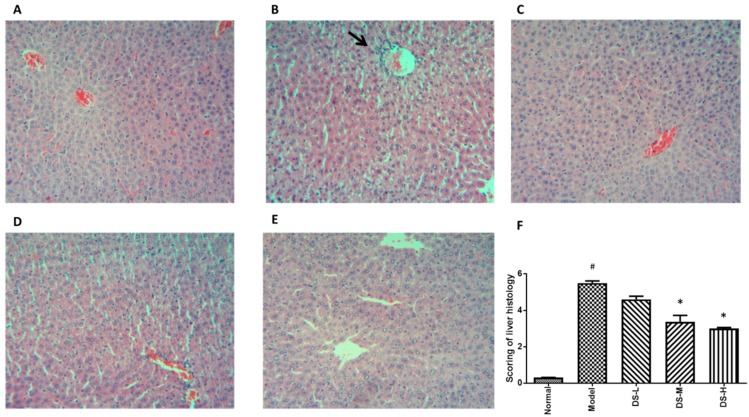
H&E staining results after Danshen treatment in acute ALD (original magnification 100×). (**A**) The normal group had a clear structure of the hepatic lobule, and there were no visible lesions; (**B**) In the model group, typical pathological characteristics such as inflammatory infiltration (arrows) can be detected; (**C**–**E**) Treatment with Danshen (0.093, 0.28, 0.84 g/kg) at 0.2 mL for nine days attenuated the inflammation in the liver; (**F**) Scoring of liver histology of acute ALD mice with Danshen treatment (Mean ± SD). DS-L for low dose of Danshen treatment group (0.093 g/kg); DS-M for middle dose of Danshen treatment group (0.28 g/kg); DS-H for high dose of Danshen treatment group (0.84 g/kg). ^#^ *p* < 0.05, compared with normal group * *p* < 0.05 when compared with model group.

Treatment with Danshen can decrease the liver weight to body weight ratio. Compared with the control group, alcohol administration slightly reduced body weight in mice, while Danshen treatment had no significant effect on body weight (Figure 1A,B). Alcohol administration exacerbated the development of liver injury in mice, as indicated by augmented liver weight to body weight ratio and increased serum AST and ALT levels. Significantly decreased ALT and AST levels were detected in acute ALD mice after Danshen treatment (Figure 1C,D). Mice liver morphology also appeared normal, accompanied with lower liver damage scoring in the Danshen treatment group. This further postulated that the liver histology results were consistent with the liver function test (Figure 2). The biochemical results showed that Danshen could increase hepatic SOD level, but had no significant influence on hepatic level of CAT or GSH-Px (Figure 1E–H). Our results also showed that administration of alcohol to mice markedly increased MDA levels compared with the normal control group. This result indicates that peroxidation progress of hepatic lipid occurred rapidly after formation of fatty liver by acute alcohol consumption. When mice were orally administered with 0.28 g/kg Danshen, the levels of MDA in hepatic tissues were significantly (*p* < 0.05) reduced by up to 31.6% of ethanol control group. These results indicate that Danshen can protect against the alcohol-induced hepatic lipid peroxidation process.

### 2.2. The Hepatoprotective Effect of Danshen on Chronic ALD

**Figure 3:**
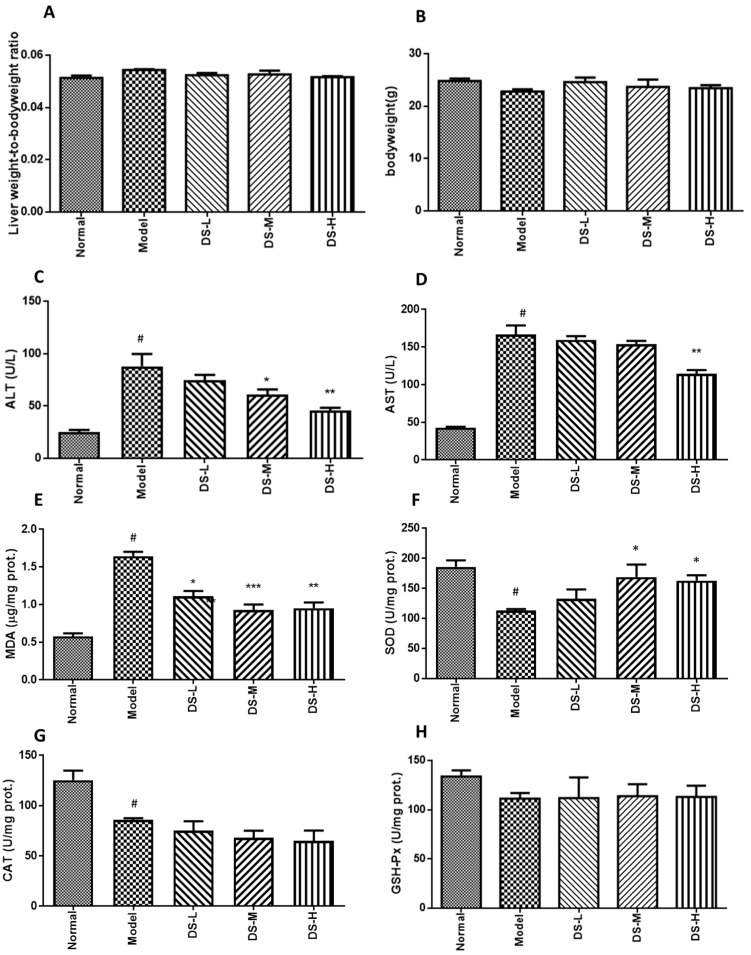
Biochemical assays results of Danshen treatment on chronic ALD (original magnification 100×). (**A**) Serum ALT level; (**B**) serum AST level; (**C**–**H**) the production of MDA and the activity of SOD, CAT, and GSH-Px in liver tissues. DS-L for low dose of Danshen treatment group (0.093 g/kg); DS-M for middle dose of Danshen treatment group (0.28 g/kg); DS-H for high dose of Danshen treatment group (0.84 g/kg). Compared with the normal group, ^#^ *p* < 0.05; compared with model group, * *p* < 0.05, ** *p* < 0.01 and *** *p* < 0.001.

**Figure 4:**
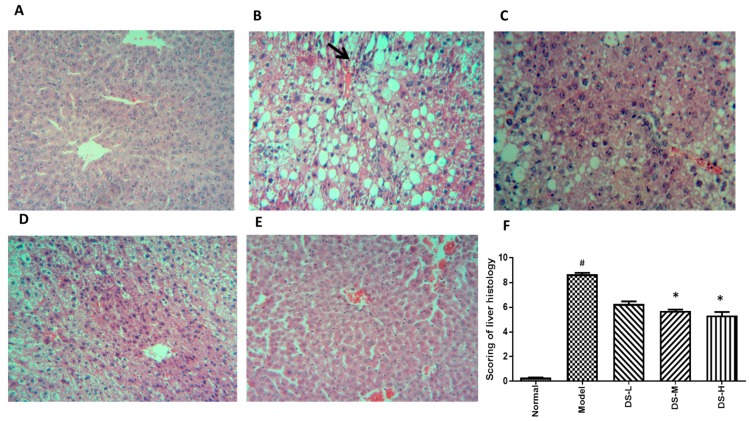
H&E staining results after Danshen treatment in chronic ALD (original magnification 100×). Treatment with Danshen for six weeks attenuated the inflammation and macrovacuolar degeneration in chronic ALD mice mode. (**A**) The normal group had a clear structure of the hepatic lobule, and there were no visible lesions; (**B**) In the model group, typical pathological characteristics such as inflammatory infiltration (black arrow) and microvacuolar degeneration can be observed; (**C**–**E**) Treatment with Danshen (0.093, 0.28, 0.84 g/kg) significantly attenuated the inflammation and vacuolar degeneration in the liver; (**F**) Scoring of liver histology of chronic ALD mice with Danshen treatment (Mean ± SD). DS-L for low dose of Danshen treatment group (0.093 g/kg); DS-M for middle dose of Danshen treatment group (0.28 g/kg); DS-H for high dose of Danshen treatment group (0.84 g/kg). ^#^ *p* < 0.05, compared with normal group * *p* < 0.05 when compared with model group.

Chronic alcohol consumption did not produce any significant (*p* < 0.05) changes in body weights of the mice under our experimental conditions as well as the liver weight to body weight ratio. Reduced serum AST and ALT levels after Danshen treatment indicated that Danshen dose-dependently reduced liver injury induced by chronic alcohol consumption (Figure 3). Histological analysis was conducted. As shown in Figure 4A, the hepatocytes and plate from hepatic tissue sample of the normal control group have intact structure, and the boundary between hepatocytes is clear. Insides of cellular structures are clean without impurities and droplets. However, the hepatocytes of chronic ALD model showed the infiltration of lymphocytes and vacuolar degenerations (Figure 4B). Co-administration of Danshen attenuated the above histopathological changes. Scoring on liver damage gave conclusions consistent with the serum AST and ALT test. Hepatic MDA level was significantly reduced in mice treated with Danshen. Hepatic level of SOD was increased after Danshen treatment, but no significant change in the hepatic level of CAT or GSH-Px was detected (Figure 3).

### 2.3. The Hepatoprotective Effect of Danshen on NAFLD

**Figure 5:**
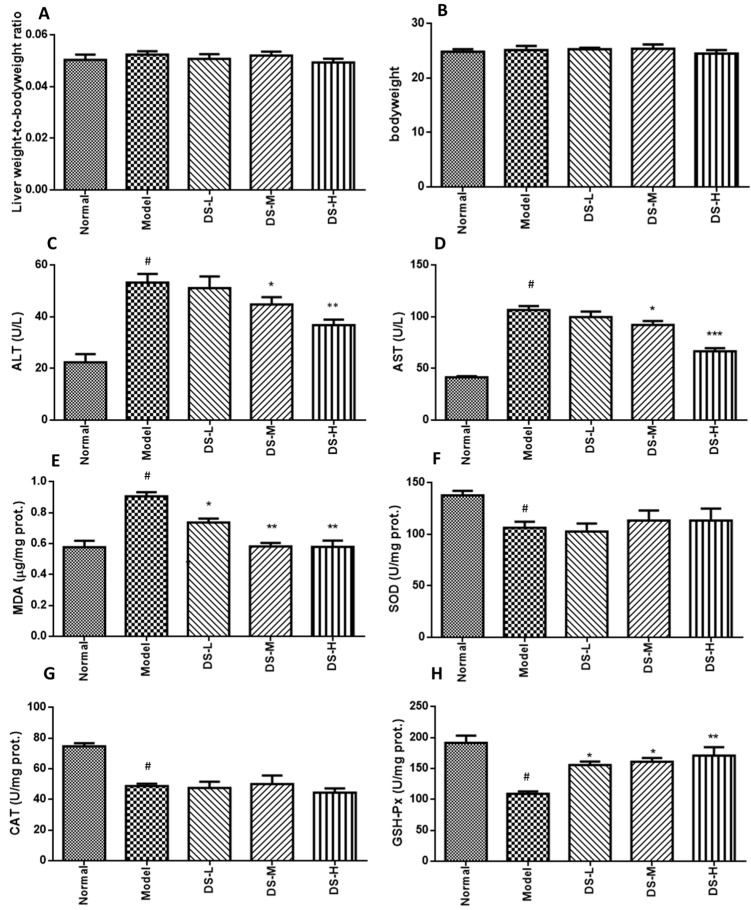
Biochemical results of Danshen treatment on mice model of NAFLD. (**A**) Serum ALT level; (**B**) serum AST level; (**C**–**H**) the production of MDA and the activity of SOD, CAT, and GSH-Px in liver tissues. DS-L for low dose of Danshen treatment group (0.093 g/kg); DS-M for middle dose of Danshen treatment group (0.28 g/kg); DS-H for high dose of Danshen treatment group (0.84 g/kg). Compared with Normal group, ^#^ *p* < 0.05; compared with model group, * *p* < 0.05, ** *p* < 0.01 and *** *p* < 0.001.

**Figure 6:**
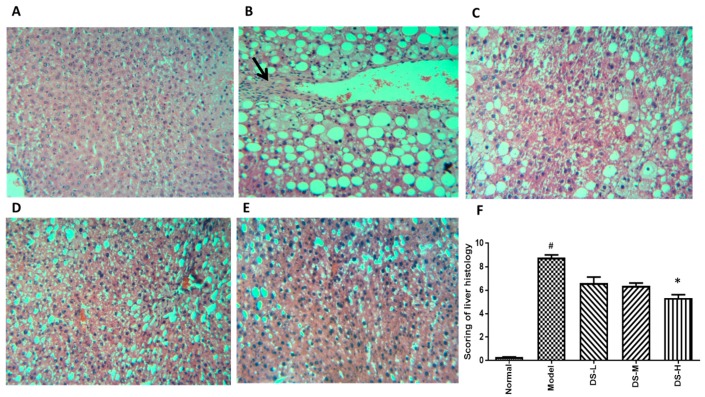
H&E staining results after Danshen treatment in NAFLD (original magnification 100×). Treatment with Danshen for six weeks attenuated the inflammation and macrovacuolar degeneration in NAFLD. (**A**) The normal group had a clear structure of the hepatic lobule, and there were no visible lesions; (**B**) In the model group, typical pathological characteristics such as inflammatory infiltration (black arrow) and macrovacuolar degeneration can be observed; (**C**–**E**) Treatment with Danshen (0.093, 0.28, 0.84 g/kg) for six weeks significantly attenuated the inflammation and macrovacuolar degeneration in the liver; (**F**) Scoring of liver histology of NAFLD mice with Danshen treatment (Mean ± SD). DS-L for low dose of Danshen treatment group (0.093 g/kg); DS-M for middle dose of Danshen treatment group (0.28 g/kg); DS-H for high dose of Danshen treatment group (0.84 g/kg). ^#^ *p* < 0.05, compared with normal group * *p* < 0.05 when compared with model group.

**Figure 7:**
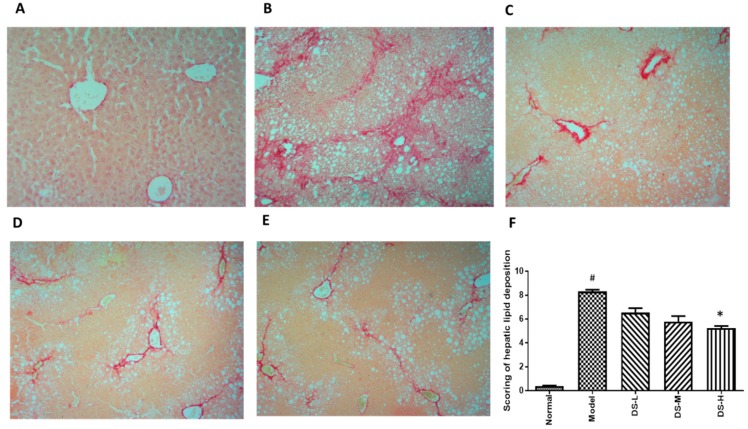
Picrosirius red staining results after Danshen treatment in NAFLD (original magnification 100×). Treatment with Danshen attenuated the collagen deposition in NAFLD. (**A**) The normal group showed little collagen deposition within hepatocytes; (**B**) in the model group, PSR staining showed significant collagen deposition level within hepatocytes; (**C**–**E**) treatment with Danshen (0.093, 0.28, 0.84 g/kg) for six weeks significantly reduced collagen deposition levels in the liver; (**F**) scoring of collagen deposition of NAFLD mice with Danshen treatment (Mean ± SD). DS-L for low dose of Danshen treatment group (0.093 g/kg); DS-M for middle dose of Danshen treatment group (0.28 g/kg); DS-H for high dose of Danshen treatment group (0.84 g/kg). ^#^ *p* < 0.05, compared with Normal group * *p* < 0.05 when compared with model group.

Treatment of Danshen could significantly reduce the AST level in a dose-dependent manner, and also slightly reduce the ALT level (Figure 5). Although AST is a more specific and sensitive indicator for liver damage in NAFLD [25,26], the results still indicate the effect of Danshen in alleviating NAFLD-induced liver damage. The H&E staining exhibited visible intracellular vacuolization that marked lipid accumulation and obvious inflammatory infiltration in the model group. However, the Danshen-treated group showed decreased levels of accumulated lipid droplets and inflammatory infiltrates. Histological scoring results showed consistent with the conclusion of serum ALT and AST levels (Figure 6). Hepatic MDA level was dose-dependently reduced while GSH-Px level was increased in mice treated with Danshen. However, a minimal effect on hepatic SOD and CAT activity could be detected after Danshen treatment (Figure 5). In addition, the PSR staining revealed a more pronounced development of fibrosis in livers of the model group mice, compared to mice that received Danshen (Figure 7).

### 2.4. In Silico-Based Network Construction and Analysis

**Figure 8:**
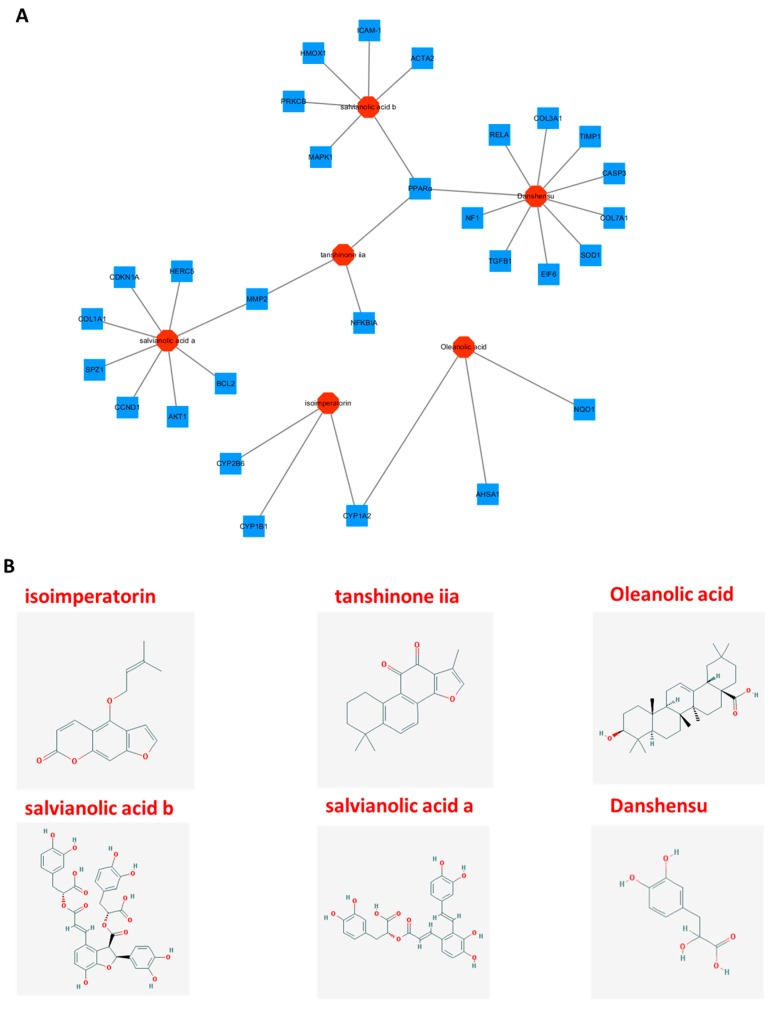
Compound–target networks and corresponding compound structures. The Compound-target network related to hepatoprotective effects in ALD and NALFD was shown in (**A**). The red octagons are active compounds from Danshen and the blue squares represent potential hepatoprotective target genes; the gray line represents the compound–target interaction; (**B**) The corresponding chemical structures of the six potential hepatoprotective components from Danshen.

The active ingredients of Danshen were predicted through in silico-based pharmacology method. To further illuminate the relationship between bioactive compounds and potential target genes, a drug-target network was built through network analysis (Figure 8). The results showed that six active compounds of Danshen were found to affect different potential targets, which may exhibit hepatoprotective effects (Table S1). Among the six active compounds, Danshensu exhibits the largest number of hepatoprotective target connections (10), followed by salvianolic acid a (8), and salvianolic acid b (6). For the 29 potential targets, the network showed peroxisome proliferator-activated receptor alpha (PPARα) had the largest number of compound interactions (tanshinone iia, salvianolic acid b and Danshensu), followed by CYP1A2 (isoimperatorin and Oleanolic acid) and MMP-2 (tanshinone iia and salvianolic acid a). The remaining 26 targets showed interactions with only one compound. Those high-degree nodes in the network, which had more target–compound interactions, are likely to play a more important role in treating ALD and NAFLD [27]. Information on 29 potential hepatoprotective targets in Danshen can be found in the Supplementary Materials (Table S1); all the data were manually collected from the TTD, PharmGKB, and CTD databases. 

## 3. Discussion

In the long history of TCM practice, Danshen has been shown to have multiple pharmacological activities, including hepatoprotective effect. Although the chemistry composition of Danshen has been extensively studied, the active ingredients and related mechanisms that contribute to its hepatoprotective activity in ALD and NAFLD are far from clear. In the present study, we demonstrated that Danshen might exhibit a preventive effect on acute and chronic ALD as well as NAFLD in a mouse model. Danshen could significantly decrease the MDA level in both ALD and NAFLD models. MDA is formed as a result of peroxidation of unsaturated fatty acids, which is the end product of lipid peroxidation. Since the MDA level is considered an indicator of polyunsaturated fatty acid damage, the decreased MDA level indicated attenuated oxidative damage in liver disease [28,29]. Previous studies showed that the detoxification pathway by the internal anti-oxidative system should be the result of an interacting network of multiple enzymes, with SOD catalyzing the first step and then CAT and GSH-Px removing hydrogen peroxide [30,31]. Although our data indicated that Danshen could increase the expression of SOD in ALD model and improve the activity of GSH-Px in NAFLD model, the overexpression of a single antioxidant enzyme in the liver is not sufficient to activate the internal anti-oxidative enzyme system and reduce the toxicity of superoxide [32]. Thus, Danshen seemed to play its anti-oxidation role through some other mechanisms in ALD and NAFLD. This postulation is partly consistent with our network pharmacological analysis results. 

Peroxisome proliferator-activated receptor alpha (PPARα), which had the largest number of compound interactions in our network study, was indicated as the key regulator of lipid peroxidation in ALD and NAFLD. Tanshinone iia, salvianolic acid b, and Danshensu might be the potential active compounds of Danshen to target PPARα and related signaling pathways. In previous studies, PPARα may mediate NAFLD through a periostin-dependent pathway. It can regulate fatty acid oxidation by activating the periostin-dependent JNK signaling pathway and further activate hepatosteatosis in vivo and in vitro [33,34,35]. Activation of PPARα is also associated with increased mitochondrial glutathione (GSH) in the liver and decreased levels of circulating fatty acyl-carnitines [36]. Furthermore, PPARα plays a protective role to enhance mitochondrial function in response to chronic alcohol consumption by adaptive transcriptional activation and we suggest that activation of this nuclear receptor may be of therapeutic value in the treatment for ALD [37]. These studies indicated that PPARα may emerge as an intracellular target of Danshen for preventing the development of ALD and NAFLD. Cytochrome P450, family 1, subfamily A, polypeptide 2 (CYP1A2), a member of the cytochrome P450 mixed-function oxidase system, may also be involved in the metabolism of xenobiotics such as ethanol in the liver. Previous research has revealed that CYP1A2 plays an important role in alcohol-induced liver steatosis by catalyzing many reactions involved in ethanol metabolism and synthesis of cholesterol, steroids, and other lipids [38,39]. CYP1A2 could produce ROS to further promote oxidative stress and inflammation. In the early stage of ALD, this enzyme can generate ROS in the liver as a consequence of alcohol exposure. ROS will further produce excessive oxygen free radicals and lead to lipid peroxidation, as well as oxidative stress damage [40,41,42]. Our in silico study results indicated that the active ingredients in Danshen, such as isoimperatorin and oleanolic acid, may regulate the expression of CYP1A2, CYP2B6, and CYP1B1, thus further attenuating oxidative damage in the liver. Furthermore, like PPARα, CYP1A2 activity is also associated with intracellular GSH concentration [43]. GSH, which is one of the most important cellular non-enzymatic antioxidants may be involved in the hepatoprotective and antioxidant activity of Danshen in ALD and NAFLD. Our findings also suggest that Danshen and its potential active compounds (tanshinone iia and salvianolic acid a) may be useful in attenuating hepatic injury in CCl4-induced liver fibrosis in NAFLD model. Matrix metalloproteinase-2 (MMP2) may play a pivotal role in the anti-fibrosis effects of Danshen according to our network study. MMP2 is important in the formation of hepatic fibrosis, degrading certain kinds of extra cellular matrix (ECM) such as collagens and proteoglycans. Downregulation of MMP2 expression and the TGF-β1/Smad signaling pathways can relieve liver fibrosis in rats [44]. MMP2 activity is also critical for TGFβ2-induced matrix contraction, which may promote fibrosis in vitro [45]. Thus, MMP2 may be an important target for tissue repairing and preventing interstitial fibrosis in NAFLD. Generally, in the current study, we found that Danshen may relieve hepatic inflammation, fatty liver, and fibrogenesis in ALD and NAFLD mice without obvious side effects. Although our studies have shown that Danshen extract and its potential active ingredients may alleviate liver diseases in mice models, these animal models cannot predict the actual effect of Danshen on liver diseases in human patients. A randomized, double-blinded, placebo-controlled clinical trial should be performed in a future study to critically examine whether Danshen has a preventive and/or therapeutic effect on ALD and NAFLD patients.

An innovation of this study is combining in vivo studies with network pharmacology research. For the multiple components/multiple targets interaction model of TCM herbal medicines, conventional experimental research faces a situation of long-term investment to investigate the complex interaction mechanisms. Thus, our network pharmacology study, which integrates the systems biology and in silico technologies, may offer a direction for the mechanistic study of hepatoprotective effects of TCM herbal medicines. The pharmacological network analysis results illustrated the potential active ingredients and mechanism of action of Danshen in the management of ALD and NALFD. Therefore, network pharmacology may be a forceful tool for exploring the potential mechanism of action of TCM herbal medicines and developing novel active ingredients. A network pharmacology-based approach combined with in vivo studies might be available for elucidating the relationship between complex diseases such as liver diseases and TCM herbal medicine interventions. Notwithstanding the advances in recent network pharmacology research, there are some crucial technical issues to be addressed and improved for data collection on herbal medicines. First of all, the inventory of herbal products remains incomplete and novel chemical structures are being discovered. Secondly, previous researchers explored and provided only a small number of hepatoprotective target genes. Last but not least, stringently assessing the relationships between compounds and corresponding targets and obtaining accurate action modes such as activated drug–target interactions or inhibited drug–target interactions are still a challenge for the present network pharmacology study [46,47]. To resolve these problems, herbal compound libraries should be established and further enriched to better correlate compound functions with structures. Furthermore, experimental verification of the potential hepatoprotective compounds after in silico screening is needed to validate the accurate interactions between drugs and proteins based on theoretical predictions.

## 4. Materials and Methods

### 4.1. Reagents

Ethanol (99.9%, Thermo Scientific, Waltham, MA USA); carbon tetrachloride (CCl_4_) (99.9%, Thermo Scientific); Liquid ethanol diet (Bio-Serv, Flemington, NJ, USA); Control liquid dextrose diet (Bio-Serv); Choline-deficiency, amino acid-defined diet (CDAA) (Research Diets); Detection Kits for alanine transaminase (ALT), aspartate transaminase (AST), malondialdehyde (MDA), superoxide dismutase (SOD), glutathione peroxidase (GPx), and catalase (CAT) (Jiancheng, Nanjing, China); Direct Red 80 (Sigma-Aldrich, St. Louis, MO, USA); Picric acid (Sigma-Aldrich); Oil Red O (Sigma-Aldrich); Danshen aqueous extracts was prepared by Vitagreen^®^ (Hong Kong, China), the root of *Salvia miltiorrhiza* Bunge was cut into small pieces and soaked for 2 h, then boiled for 1 h under a medium-heat fire after first being boiled under a high-heat fire. After being filtered, the residue was boiled again using the same method. All filtrate was collected, combined and then concentrated at 60 °C to a final concentration of 0.5 g/mL.

### 4.2. Animal Models and Treatments

For the alcohol-induced acute liver injury model, male C57BL/6J mice (four weeks of age) in treatment group were given three doses of Danshen extracts (0.093, 0.28, 0.84 g/kg/day) via gavage for nine days. Normal and Model groups of mice received equal volume (0.2 mL) of saline water. 6 h after the last treatment, the model group and treatment group of mice received ethanol (6 g/kg via gavage). The normal group of mice received the same volume of isocaloric/isovolumetric maltodextrin solution. This model was slightly modified from Enomoto et al. [48]. After 12 h of ethanol treatment, mice were euthanized by cervical sacrificed, and serum and liver samples were collected. For the induction of chronic ALD model, mice were initially fed the control liquid dextrose diet for three days to acclimate them to the liquid diet. Afterward, the mice were fed either the liquid ethanol diet or the control liquid dextrose diet for two weeks, as described by previous studies [49,50]. For the treatment group, mice were given Danshen extracts at different dose (0.093, 0.28, 0.84 g/kg) via gavage daily throughout the experiment. Normal and model groups of mice received equal volume (0.2 mL) of saline water. Twelve hours after the last treatment, mice were euthanized by cervical sacrificed, and serum and liver were collected. To induce chronic NAFLD model, we introduced the CDAA diet as described by Fujita et al. [51]. A low dose of CCl_4_ (0.4 μL/g body weight, twice/week) was used as promoter of fibrosis. Mice were fed with control chow or CDAA chow for six weeks in this study. Then, three doses of Danshen (0.093, 0.28, 0.84 g/kg) were given to the treatment group through oral administration every day. Normal and model groups of mice received an equal volume (0.2 mL) of saline. At the end point, mice were euthanized by cervical dislocation; serum and liver were collected for further analysis. All experimental protocols involving mice were approved by the Committee on the Use of Live Animals in Teaching and Research (CULATR) of the University of Hong Kong. CULATR number: 3637-15. Project Start Date: 27 March 2015. Animal license was approved by Department of Health, Hong Kong Special Administrative Region Government. Animal license number: (13-671) in DH/HA&P/8/2/3 Pt.54. Approved date: 13 December 2013.

### 4.3. Biochemical Assays

Serum samples were separated by centrifugation at 3000 rpm for 10 min and were kept at −20 °C until analysis. The serum levels of ALT, AST were determined with commercial kits. All of the procedures were carried out according to the manufacturers’ instructions. Liver tissue was homogenized in lysis buffer, centrifuged at 10,000× *g* for 5 min at 4 °C, and the supernatants were collected. The activity of GSH-Px, SOD, CAT and the production of MDA were measured according to the manufacturers’ instructions.

### 4.4. Liver Histology

Formalin-fixed tissues were stained with hematoxylin and eosin (H&E) and analyzed by microscopy. The liver damage score was measured by three individual examiners with the following criteria, 0: no damage; 1–3: mild damage; 4–6: intermediate damage; 7–9: severe damage; 10: destruction of liver structure [52]. In addition, for assessing liver fibrosis, we also conducted Sirius-Red staining in hepatic tissue biopsy. Scoring on the stained sections was made by three individual examiners with the following criteria, 0: no signs of observed fibrosis. 1–3: no extension of portal area fibrosis. 4–6: fibrosis occurring in the portal area with an intact lobule structure. 7–9: fibrosis associated with a broken lobule structure, but no signs of cirrhosis. 10: fibrosis and the formation of cirrhosis [53]. All the stained sections were observed and photographed under a microscope (with 100× magnification).

### 4.5. Network Pharmacology Analysis

#### 4.5.1. Molecular Database Construction

Chemical ingredients of Danshen were manually collected from related literature and two phytochemical databases: Traditional Chinese Medicine Systems Pharmacology Database (TCMSP) (Available online: http://lsp.nwsuaf.edu.cn/) and TCM Database@Taiwan (Available online: http://sm.nwsuaf.edu.cn/lsp/tcmsp.php) [31,32,33,34,35,36].

#### 4.5.2. Pharmacokinetic ADME Evaluation

In this step, an in silico integrative model—ADME was used to select the ingredients with favorable pharmacokinetics properties. The ADME system used in this study including PreOB (predict oral bioavailability) and PreCaco-2 (predict Caco-2 permeability). Oral bioavailability (OB) is one of the most vital pharmacokinetic properties of orally administered drugs as it plays an important role for the efficiency of the drug delivery to the systemic circulation [54,55]. Here, a reliable in silico screening model OBioavail 1.1 was employed in OB value calculation of the constituents in Danshen. This model was constructed based on 805 structurally diverse drugs and drug-like molecules. Multiple linear regression, partial least square and support vector machine methods were applied during this model building, ending up with determination coefficient (*R*^2^) = 0.80 and standard error of estimate (*SEE*) = 0.31 for test sets. [56,57]. In addition, for orally administered drugs, another pivotal problem is their movement across the intestinal epithelial barrier, which determines the rate and extent of human absorption and ultimately affects its bioavailability [58]. Thus, a preCaco-2 model was used to predict the efficiency of drug absorption. The phytochemical information of the compounds with their Caco-2 permeability properties were explored using the TCMSP database; detailed parameters information, screening criteria, and calculations can be obtained from TCMSP website (Available online: http://sm.nwsuaf.edu.cn/lsp/load_intro.php?id=29). Finally, compounds with OB ≥ 33% and Caco2 ≥ 0.4 cm/s were regarded as candidate ingredients for further study. It is worth noting that the OB value of salvianolic acid A and salvianolic acid B are lower than 33%, but both of them are widely expected to exhibit hepatoprotective effects in vitro and in vivo [11,59,60,61]. Thus, these two compounds were also regarded as candidate compounds for further analysis.

#### 4.5.3. Identification of Associated Proteins and Genes

The integrative efficacy of the ingredients in Danshen was determined by analyzing the compounds and targets interactions obtained from the Search Tool for Interactions of Chemicals and Proteins (STITCH) database(Available online: http://stitch.embl.de/) [44], HIT (Herbal Ingredients’ Targets) Database (Available online: http://lifecenter.sgst.cn/hit/) [45] and omics-based Ligand-Target Chemogenomic model (LTC) [46], respectively. Then, for better defining the role of Danshen in hepatoprotective potentials, these targets were mapped to the Therapeutic Target Database (TTD, Available online: http://bidd.nus.edu.sg/group/ttd/) [47], PharmGKB (Available online: http://www.pharmgkb.org) [48], and the Comparative Toxicogenomics Database (CTD, Available online: http://ctdbase.org/) [49] to eliminate the unrelated target protein and provide a more complete and accuracy view on compound-hepatoprotective target associations. The targets with hepatoprotective potentials were retrieved from TTD, PharmGKB and CTD database by using the following search terms: liver disease, hepatic damage, alcoholic liver, non-alcoholic liver, fatty liver, liver fibrosis, oxidative damage, steatosis, oxidative damage, and lipid metabolism. 

#### 4.5.4. Network Construction

**Figure 9:**
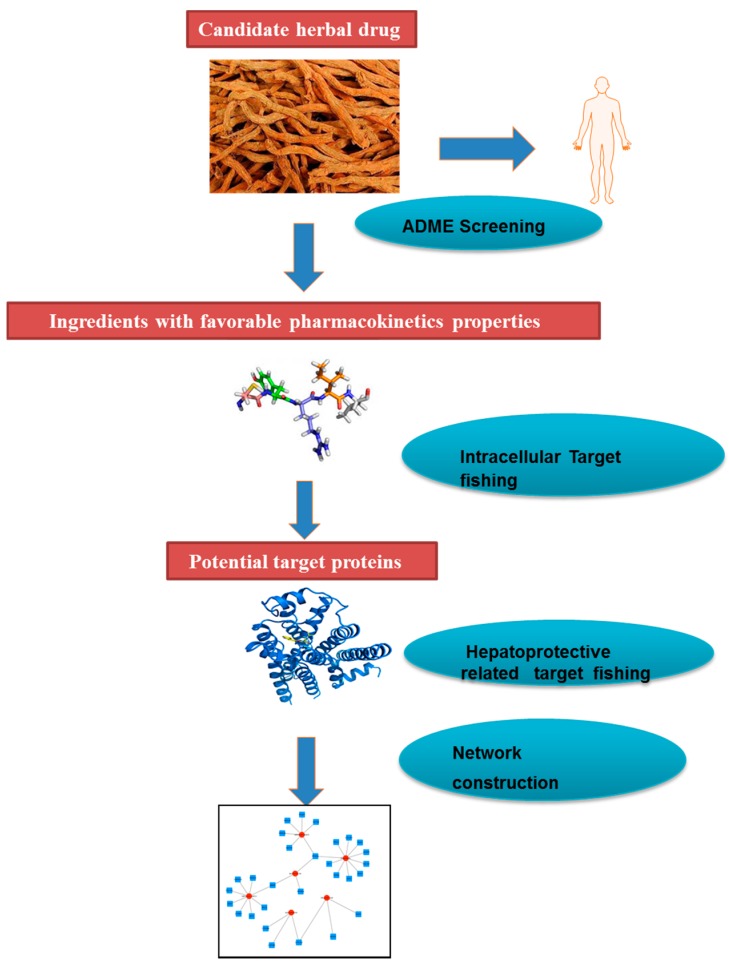
A flowchart to schematically describe the network pharmacology process.

To further probe the relationships between the compounds and targets associated with hepatoprotective effects, the Compound–Target network plotting was generated by Cytoscape 3.4.0 (Available online: http://www.cytoscape.org/) [50]. In the graphical network plot, nodes represent the compounds or proteins, and edges encode the compound–target interactions. In order to specify the importance of a node and how this node influences the communication between two nodes, all the properties of the network were analyzed using Network Analysis plugin. A flowchart to schematically describe the network pharmacology process in this study is shown in Figure 9.

### 4.6. Statistical Analysis

All the data are presented as the mean ± SD and analyzed by one-way analysis of variance using SPSS17 software (SPSS Inc., Chicago, IL, USA). *p* < 0.05 was considered statistically significant.

## 5. Conclusions

Our studies suggested that Danshen could alleviate hepatic inflammation, fatty degeneration, and haptic fibrogenesis in ALD and NAFLD models. The combination of network pharmacology strategy with in vivo experimental study may provide a forceful tool for exploring the mechanism of action of traditional Chinese medicine herb and developing novel bioactive ingredients.

## Supplementary Materials

Supplementary materials can be found at www.mdpi.com/1422-0067/18/3/620/s1.
