# Supplementary material for: A Biomedical Investigation of the Hepatoprotective Effect of Radix salviae miltiorrhizae and Network Pharmacology-Based Prediction of the Active Compounds and Molecular Targets
Source: Int J Mol Sci. 2017 Mar 13;18(3):620. doi: 10.3390/ijms18030620 (PMC5372635; doi:10.3390/ijms18030620)
Supplement: Supplementary file 1 [file ijms-18-00620-s001.pdf]

Table S1. The information of 29 potential hepatoprotective targets in Danshen.

Target Gene	Target Protein	Organism	Roles in Hepatoprotective Effects
CYP1A2	cytochrome P450, family 1, subfamily A, polypeptide 2	Homo sapiens	Oxidizes a variety of structurally unrelated compounds, including steroids, fatty acids
CYP2B6	cytochrome P450, family 2, subfamily B, polypeptide 6	Homo sapiens	Oxidizes a variety of structurally unrelated compounds, including steroids, fatty acids
CYP1B1	cytochrome P450, family 1, subfamily B, polypeptide 1	Homo sapiens	Oxidizes a variety of structurally unrelated compounds, including steroids, fatty acids
MMP2	matrix metallopeptidase 2	Homo sapiens	Tissue repair and induce interstitial fibrosis
PPARα	peroxisome proliferator-activated receptor alpha	Homo sapiens	Key regulator of lipid metabolism
NFKBIA	nuclear factor of kappa light polypeptide gene enhancer in B-cells inhibitor, alpha	Homo sapiens	On cellular stimulation by immune and proinflammatory responses
AHSA1	activator of heat shock 90 kDa protein ATPase homolog 1	Homo sapiens	Involve in Grb2-p38 MAPK signaling pathway in fibrosis
CYP1A2	cytochrome P450, family 1, subfamily A, polypeptide 2	Homo sapiens	Oxidizes a variety of structurally unrelated compounds, including steroids, fatty acids
NQO1	NAD(P)H dehydrogenase, quinone 1	Homo sapiens	Involve in alcohol detoxification pathways
HMOX1	heme oxygenase (decycling) 1	Homo sapiens	Alleviate liver inflammation and reduced oxidative stress
ICAM-1	intercellular adhesion molecule 1	Homo sapiens	Mediate adhesive interaction in fibrosis process
MAPK1	mitogen-activated protein kinase 1	Homo sapiens	Regulate cytoskeletal rearrangements in fibrosis process
PRKCB	protein kinase C, beta	Homo sapiens	Regulate oxidative stress-induced cell damage
ACTA2	actin, alpha 2, smooth muscle, aorta	Homo sapiens	Involve in myofibroblast cell motility during wound healing in liver
SPZ1	spermatogenic leucine zipper 1	Homo sapiens	The transcriptional factors of liver fatty

			acid binding protein
COL1A1	collagen, type I, alpha 1	Homo sapiens	Transcriptional repressor of the collagen
BCL2	B-cell CLL/lymphoma 2	Homo sapiens	Regulate the response to mitochondrial damage and related oxidative damage
CCND1	cyclin D1	Homo sapiens	Functions as a mediator of β-catenin during hepatocarcinogenesis
HERC5	HECT and RLD domain containing E3 ubiquitin protein ligase 5	Homo sapiens	Acts as a positive regulator of innate antiviral response in liver cells
AKT1	v-akt murine thymoma viral oncogene homolog 1	Homo sapiens	Regulate lipid metabolism
CDKN1A	cyclin-dependent kinase inhibitor 1A	Homo sapiens	Regulate hepatic cell cycle in hepatocarcinogenesis
EIF6	eukaryotic translation initiation factor 6	Homo sapiens	Regulate hepatocarcinogenesis by mediating cellular response to DNA damage.
CASP3	caspase 3	Homo sapiens	Apoptosis inhibitory protein in hepatocarcinogenesis
COL7A1	collagen, type VII, alpha 1	Homo sapiens	Regulate fibrosis by impacts on extracellular matrix (ECM) proteins such as type IV collagen
COL3A1	collagen, type III, alpha 1	Homo sapiens	Regulate fibrosis by impacts on extracellular matrix (ECM) proteins such as type IV collagen
TGFB1	transforming growth factor, beta 1	Homo sapiens	Regulate liver cancer cells proliferation
TIMP1	TIMP metallopeptidase inhibitor 1	Homo sapiens	Tissue repair and induce interstitial fibrosis
SOD1	superoxide dismutase 1	Homo sapiens	Destroys radicals which are normally produced within the cells, such as oxidants
RELA	v-rel reticuloendotheliosis viral oncogene homolog A	Homo sapiens	Involve in hepatic inflammation
